# The role of *SYNE1/2* variants as a potential predisposition factor for the onset of endometriosis

**DOI:** 10.3389/fimmu.2026.1888334

**Published:** 2026-08-25

**Authors:** Aurora Santin, Silvia Pegoraro, Miriam Toffoli, Giulia Pianigiani, Andrea Balduit, Gabriella Zito, Alessandro Mangogna, Paola Trevisiol, Lara Emily Rosso, Federica Mannino, Giovanni Di Lorenzo, Francesca Rossi, Eva Andreuzzi, Chiara Agostinis, Giuseppe Ricci, Roberta Bulla, Giorgia Girotto

**Affiliations:** 1 Department of Medicine, Surgery and Health Sciences, University of Trieste, Trieste, Italy; 2 Institute for Maternal and Child Health - IRCCS “Burlo Garofolo”, Trieste, Italy; 3 Department of Life Sciences, University of Trieste, Trieste, Italy; 4 Institute of Pathological Anatomy, Department of Medicine, University of Udine, Udine, Italy

**Keywords:** endometriosis, migration, Nesprin, *SYNE2*, whole-exome sequencing

## Abstract

Endometriosis (EM) is a chronic, inflammatory gynaecological disorder defined by the presence of endometrial-like tissue outside the uterine cavity, most frequently affecting the ovaries, peritoneum, and uterosacral ligaments. Despite its prevalence and the significant impact on life quality, EM is often underdiagnosed, with an average delay of about nine years, particularly affecting adolescents and young women. The complex aetiology involves genetic, environmental, and immune factors, with whole-exome sequencing (WES) emerging as a potential tool for identifying relevant genetic variants. Research indicates that innate immune dysfunction, mechanotransduction, and epithelial-to-mesenchymal transition promote endometrial cell migration and lesion formation, processes regulated by nuclear envelope integrity and cytoskeletal dynamics. The LInker of Nucleoskeleton and Cytoskeleton (LINC) complex, specifically Nesprin-1 and Nesprin-2, encoded by *SYNE1* and *SYNE2*, is crucial for these processes. Genome-wide studies have linked *SYNE* genes to EM risk, showing downregulation in affected patients, and rare variants in these genes have been identified, though their functional implications are still unclear. To this purpose, WES was performed on 204 EM patients to identify rare (MAF <0.1%), damaging variants in SYNE1/2. Primary endometriotic cells (EMCs) were isolated from ovarian lesions of variant carriers (n=4) and wild-type (WT) non-carrier controls (n=4). Functional characterization included somatic WES, RT-qPCR, Western blot, confocal immunofluorescence, and Transwell migration assays. WES identified 11 rare, likely damaging *SYNE1/2* variants in 12 patients. Immunofluorescence revealed a distinct protein mislocalization, WT EMCs displayed physiological Nesprin-2 confinement at the nuclear envelope, whereas variant carriers exhibited a diffuse cytoplasmic distribution polarized along actin stress fibres. We demonstrated that *SYNE1/2* mutated EMCs had a markedly higher migratory capacity compared to WT controls. Here, *in vitro* experiments demonstrated, for the first time, the involvement of Nesprin-2 in endometrial cell migration, supporting a mechanistic link between nuclear–cytoskeletal disruption and the invasive phenotype of endometriotic cells (EMCs). These findings provide new insights into EM pathogenesis and highlight *SYNE2* as a promising molecular marker for improved diagnosis and disease management.

## 1 Introduction

Endometriosis (EM) is a chronic, oestrogen-driven, inflammatory gynaecological condition characterised by the presence of endometrial-like tissue and stroma outside the uterine cavity, most commonly affecting the ovaries, peritoneum, and uterosacral ligaments (1). The delay in the diagnosis of EM, currently estimated at approximately nine years, has emerged as a significant public health concern. Timely identification and intervention for EM in adolescents and young women is crucial for improving quality of life and preventing potential long-term complications (2). Various subtypes and classification methods have been developed to describe the heterogeneity and complexity of EM, which is influenced by genetic, hormonal, immune, and environmental factors. The heterogeneous nature of EM has required a classification approach as universal and easily applicable as possible. Several systems have been proposed to assess the type and extent of the disease, but there is still no gold standard. Current classifications fail to fully capture important aspects such as pain severity, fertility outcomes, or the overall impact on quality of life (3). In particular, the most widely used system is the revised American Society for Reproductive Medicine (rASRM) scoring system, which distinguishes four stages based on a cumulative score that is calculated according to the extent and depth of lesions: I or minimal, II or mild, III or moderate, and IV or severe (4).

Although the etiopathogenesis of EM remains poorly elucidated, accumulating evidence suggests that it has a multifactorial origin, with both genetic predisposition and environmental impact contributing to disease onset and progression. Family and twin-based studies indicate a substantial heritable component, with approximately 26% of disease susceptibility attributable to common genetic variants (5, 6). Interestingly, the pathogenesis of EM is strongly associated with immune dysfunction, particularly involving innate immunity, which includes the complement system (7–9), natural killer (NK) cells, macrophages, and mast cells (10). Genetic variants or polymorphisms affecting immune-related genes may contribute to immune dysregulation, posing significant challenges to treatment efforts. This condition highlights the critical need for comprehensive genetic investigations of innate immunity to support the development of personalized medicine approaches in EM.

Over the past decade, genome-wide association studies (GWAS) have identified more than 40 loci associated with EM, implicating genes mostly involved in sex hormone signalling (*e.g.*, *ESR1*, *FSHB*), cellular proliferation (*e.g.*, *WNT4*), and migration (*e.g.*, *SYNE1*) (11). However, these common variants account for only a small fraction of EM heritability, pointing to the existence of additional genetic contributors, particularly rare variants with potentially greater biological effects (12, 13).

In this context, Whole-exome sequencing (WES) is a powerful tool for investigating such variants, enabling the detection of rare, potentially damaging germline variants within the protein-coding regions of the genome. Although only a limited number of studies have applied WES to EM (14–16), these efforts have begun to scratch the surface of the elusive genetic landscape of EM, offering a critical step towards uncovering novel molecular drivers and potential biomarkers (15).

Recent studies have highlighted the roles of mechanotransduction and epithelial/endothelial-to-mesenchymal transition in promoting endometrial cell migration and invasion, which are key processes in the establishment and progression of endometriotic lesions (17–19). These processes are strictly dependent on the nuclear envelope structure and cytoskeletal dynamics, which regulate not only cell shape and motility but also chromatin organisation, gene expression, and cellular responses to mechanical stimuli. In this context, the LInker of Nucleoskeleton and Cytoskeleton (LINC) complex, located at the nuclear envelope, is an essential player for nuclear shape and mechanical signalling, since it promotes physical connections between actin, microtubules, intermediate filaments, and the nucleus. The LINC complex is composed of SUN (Sad1 and UNC-84)-domain proteins at the inner nuclear membrane, and KASH (Klarsicht, ANC-1, Syne homology)-domain proteins, such as Nesprins, at the outer nuclear membrane (20).

Nesprins comprise a family of four members, namely Nesprin1-4; among these, Nesprin-1 and Nesprin-2 proteins, encoded by the *SYNE1* and *SYNE2* genes, have recently emerged as compelling candidates in EM. Specifically, a GWAS meta-analysis reported that the *SYNE1* gene was associated with EM risk (21), and both *SYNE1* and *SYNE2* have been reported to be downregulated in the endometrium of EM patients (22, 23).

Notably, *SYNE1* downregulation has also been observed in gynaecologic malignancies and is associated with increased nuclear malleability, a feature that may facilitate cell migration (22). Given that *SYNE2* belongs to the same gene family and shares similar structural domains and functional roles in nuclear-cytoskeletal coupling, *SYNE2* may contribute similarly to the invasive properties of endometrial cells. However, to date, no studies on EM are available.

Furthermore, our previous WES study identified rare, likely damaging variants within *SYNE1* and *SYNE2* among EM patients, thereby supporting their potential role as novel candidates for EM (15). However, the functional impact of these rare high-impact variants, particularly those affecting the underexplored *SYNE2* gene, on endometrial cell migration and proliferation remains unknown and warrants deeper investigation.

Hence, to deepen the functional consequences of these variants, we performed *in vitro* experiments, and, for the first time, we demonstrated that these genes are involved in endometrial cell migration, supporting a mechanistic link between nuclear-cytoskeletal disruption and the invasive phenotype characteristic of endometriotic lesions. Overall, investigating the role of these Nesprin proteins in the context of EM is crucial for advancing our understanding of the molecular mechanisms driving disease development and paving the way for the identification of novel molecular markers to improve diagnosis and clinical management.

## 2 Materials and methods

### 2.1 Ethical statement

All patients signed a written informed consent form to participate in the study and to allow the collection of biological samples for research purposes. The study was carried out in accordance with the Declaration of Helsinki and received approval from the Ethics Committee of the Friuli-Venezia Giulia region (Italy) (Protocol No. 47846, dated 20.12.2022; protocol number for eutopic endometrium collection: CEUR-2020-Os-220).

### 2.2 Workflow of the study: patient recruitment and sample collection

A total of 204 adult women with a surgically or clinically confirmed diagnosis of EM were enrolled at the IRCCS “Burlo Garofolo” Hospital (Trieste, Italy). Specifically, 80 of these patients were already described in our previous study by Santin et al. (15).

The EM stage was determined following the revised American Society for Reproductive Medicine (rASRM) classification for surgical patients (4), and the American Association of Gynaecologic Laparoscopists (AAGL) classification for non-surgical patients (24), allowing stratification of patients based on disease severity. For each patient, a peripheral blood sample was collected for WES analysis. In addition, for patients undergoing surgery for ovarian endometrioma, a tissue biopsy was collected whenever possible. The endometrial eutopic tissue was obtained from patients undergoing hysterectomy for leiomyoma (25).

### 2.3 WES germline samples analysis, *SYNE1* and *SYNE2* variants selection

A peripheral blood sample from each EM patient was collected in EDTA. Genomic DNA was extracted from the samples using the QIAsymphony^®^ SP instrument with QIAsymphony^®^ Midi Kit (Qiagen, Venlo, The Netherlands). DNA quality was assessed with Qubit DNA Broad Range kit (Thermo Fisher Scientific, Cat. No. Q33266, Waltham, MA, USA).

WES was carried out using an Illumina NextSeq 550 instrument (Illumina Inc., San Diego, CA, USA) with the Twist Exome 2.0 plus Comprehensive Exome Spike-in kit (Twist Bioscience, South San Francisco, CA, USA), according to the manufacturer’s protocol. The detailed WES pipeline, including library preparation, sequencing, and secondary/tertiary analyses, as well as the variant calling and filtering strategy, has been previously described (15).

For the purpose of the present study, we focused specifically on variants in the *SYNE1* and *SYNE2* genes. Variants were selected based on the following criteria: 1) Quality score >20 and Variant Allele Frequency (VAF) >30%; 2) Minor Allele Frequency (MAF) <0.1% in the general population according to the gnomAD database (https://gnomad.broadinstitute.org); 3) Predicted to be likely damaging or damaging by at least three out of four *in silico* tools, where available: CADD (26), PolyPhen-2 (27), SIFT (28), and PaPI (29).

### 2.4 Primary cell isolation and culture

Functional assays to understand the potential contribution of *SYNE1* and *SYNE2* variants were performed on cells isolated from ovarian lesion biopsies (EndoMetriotic Cells, EMCs, **Supplementary Table 1**) of EM patients or from healthy endometrium (StroEND). In our cohort, only a subset of patients underwent surgery for cyst removal, and among these, only a limited number presented lesions of sufficient size to enable the cell isolation procedure. For this reason, only four populations of EMCs derived from carrier patients (three for *SYNE2* and one for *SYNE1*), named *SYNE1/2* mut EMCs, were tested. For comparison, we selected four EMC populations from non-carrier patients to serve as wild-type (WT) controls (named WT EMCs). All EMCs were isolated from ovarian lesions as previously described by Agostinis et al. (30).

Briefly, endometriotic cysts or normal endometrial biopsies (from leiomyoma patients) were digested overnight (ON) at 4 °C with 0.25% trypsin (Sigma-Aldrich), 50 μg/mL DNase I (Roche, Milan, Italy) in PBS, and then treated with collagenase type I (3 mg/mL; Worthington Biochemical) for 30 min at 37 °C. After digestion, the reaction was stopped with 10% FBS, and the undigested tissue was removed by filtering with a 100 µm filter. Cells were maintained in Human Endothelial serum-free basal Medium (HESFM, Life Technologies, Monza, Italy), supplemented with 20 ng/mL bFGF (basic Fibroblast Growth Factor), 10 ng/mL EGF (Epidermal Growth Factor), and 10% *v/v* FBS (all from Life Technologies) at 37 °C, 5% CO_2_ in gelatin-coated flasks. StroEND were isolated and grown as described by Saxena and colleagues (25). All experiments were performed within the seventh culture passage.

### 2.5 Flow cytometry analysis for primary cell characterization

For intracellular staining, EMCs (5 x 10^5^) were fixed in 3% *v/v* paraformaldehyde (PFA) in the dark for 15 min. Next, anti-human vimentin (1:50, Sigma-Merck) and anti-human CK8/18 (1:50, Dako) primary antibodies were incubated for 45 min at 4 °C, diluted in Saponin (Farmitalia Carlo Erba, Milan, Italy) to allow permeabilization. Incubation with Alexa Fluor 488-conjugated anti-mouse and anti-rabbit secondary antibodies (1:300 in Saponin, Jackson ImmunoResearch) was performed for 30 min on ice in the dark. Cells were resuspended and fixed in 1% PFA. For membrane staining, EMCs were incubated with anti-human CD31-Alexa Fluor 488, anti- human CD90- PE, and anti-human CD45-Alexa Fluor 488 (Thermo Fisher), diluted in PBS + 1% FBS and incubated 45 min on ice. Cells were resuspended and fixed in 1% PFA. Fluorescence was acquired using the Attune NxT Flow Cytometer (Thermo Fisher) equipped with a 488 nm laser, and analysed with the FlowJo Software v5.3.0.

### 2.6 WES analysis of somatic tissue biopsy samples

DNA was extracted from EMCs using the QIAsymphony^®^ SP instrument and the QIAsymphony^®^ Midi Kit (Qiagen, Venlo, The Netherlands). DNA quantity and quality were assessed using the Qubit™ DNA Broad Range Assay Kit (Thermo Fisher Scientific, Cat. No. Q33266, Waltham, MA, USA).

For all the 204 patients, WES was performed on the Illumina NextSeq 550 platform (Illumina Inc., San Diego, CA, USA) using the Twist Exome 2.0 kit supplemented with the Comprehensive Exome Spike-in panel (Twist Bioscience, South San Francisco, CA, USA). Sequencing data processing, variant calling, and downstream analyses were conducted according to the pipeline described in Section 2.3 (“WES Germline Analysis and *SYNE1/SYNE2* Variant Selection”). Additionally, to evaluate confounding genetic factors potentially influencing the migratory phenotype of the isolated cells, a targeted *in silico* analysis was performed on the somatic WES data (encompassing both WT controls and *SYNE1/2* variant carriers). Specifically, a curated panel of 22 protein-coding genes associated with the term “endometrial cell migration,” as extracted from the GeneCards database (https://www.genecards.org/), was employed (**Supplementary Table 2**).

### 2.7 Cell lines

12Z endometriotic cell line was obtained from American Type Culture Collection (ATCC) and cultured in HESFM supplemented with 10% *v/v* FBS. The medium was supplemented with 100 U/mL penicillin and 100 μg/mL of streptomycin.

### 2.8 Gene expression analysis

EMCs, endometrial stromal cells (StroEND), and 12Z cells were lysed in RL Buffer, and RNA extraction was performed with the Total RNA Purification Kit (Norgen Biotek Corp.) following the manufacturer’s protocol. RNA was quantified with the NanoReady spectrophotometer (Tecan) and reverse transcribed with the Ultrascript^®^ 2.0 cDNA Synthesis kit (PCR Biosystems). RT-qPCR was carried out with PowerUp™ SYBR™ Green Master Mix (Applied Biosystems) using Corbett Rotor-Gene™ 6000 (Qiagen). Specific primers were used and the sequences are reported in **Supplementary Table 3**. Relative gene expression was calculated by ΔCt method, using the housekeeping gene *RPS18* for normalization, and data were expressed as 2^-ΔCt.

### 2.9 Immunofluorescence and intensity profile analysis

EMCs were grown on gelatin-coated round glass coverslips (Ø 13mm) until confluence and were fixed with 3% PFA for 15 min at RT in the dark. Cells were washed twice with PBS + 0.1% Tween and then incubated with PBS + 1% BSA + 0.1% Triton X-100 + 50mM glycine for 30 min at RT to perform blocking, quenching and permeabilization simultaneously. After washing, primary antibodies for Lamin A/C (Abcam ab232730, mouse anti-human Lamin A/C, 1:1000) and the C-terminal domain (Thermo Fisher Scientific PA5-78438, rabbit anti-human Nesprin, 1:100) of Nesprin-2 protein were added in PBS + 2% BSA for 1 h at RT. Cells were then washed twice and incubated with the secondary antibodies, anti-mouse-AlexaFluor647 (1:1000) or anti-rabbit-Cy3 (1:300) in PBS + 2% BSA, and with phalloidin-FITC (Invitrogen F432, 1:30) for 30 min at RT in the dark. DAPI was added in the last 5 min of the incubation (1:5000). Coverslips were washed twice and mounted on glass slides with a drop of ProLong™ Antifade Mountant. Images were acquired using the Zeiss LSM 900 confocal laser-scanning microscope with ZEN Microscopy Software and processed using ImageJ. The fluorescence intensity profile analysis of Lamin A/C and Nesprin-2 was assessed by Plot Profile function in ImageJ. On composite images, a line was manually drawn across the nucleus, extending into the adjacent cytoplasm on both sides, following the stress fibres orientation. For each fluorescence channel, the intensity profile along the drawn line was obtained by grey-value intensity as a function of distance (µm). The exported intensity values were plotted using GraphPad Prism 10.0 to generate overlaid fluorescence intensity (Lamin A/C in green and Nesprin-2 in red).

### 2.10 Relative migration assay

EMCs were detached with trypsin, washed with PBS, and stained with the cellular stain FastDiI™ (1:100 in PBS) for 15 min at 37 °C. The labelled cells were then washed with PBS, resuspended in serum-free HESFM, and seeded in Transwell Fluoroblok™ inserts (2.5 x 10^5^ cells/Transwell). In the well beneath the plate, either serum-free HESFM or HESFM + 10% FBS was added, serving as negative and positive controls for migration, respectively. A standard curve was created by seeding cells directly in the lower chamber of the transwell system (100% migration) or by inserting the medium exclusively without cells (0% migration) to assess autofluorescence. The fluorescent signal (excitation at 535 nm, emission at 595 nm) was detected with the Tecan SparkControl™ plate reader at the time of seeding, after 1 hour and after overnight incubation.

### 2.11 Western blot

For protein extraction, EMCs from two patients carrying *SYNE2* variants (EMC22 and EMC26) or *SYNE2*-WT patients (EMC31, EMC52, EMC33, and EMC45) were harvested and lysed in a 2× lysis buffer consisting of 4× Laemmli sample buffer (Bio-Rad), supplemented with β-mercaptoethanol, and Mammalian Protein Extraction Reagent (M-PER, Thermo Fisher Scientific) at a 1:1 ratio. Lysates were homogenized using QIAshredder columns (QIAGEN), and protein concentration was determined using the Nanodrop One (Thermo Fisher Scientific). Equal amounts of protein were resolved on a 4-20% SDS-PAGE gel (Bio-Rad) and transferred onto PVDF membranes. Membranes were blocked for 1 h in 5% non-fat dry milk in TBS-T, followed by overnight incubation at 4 °C with primary antibodies. After three washes with TBS-T, membranes were incubated for 1 h at RT with horseradish peroxidase (HRP)-conjugated secondary antibodies. Signal detection was performed using Clarity Western ECL Substrate (Bio-Rad), and images were acquired with a ChemiDoc MP imaging system (Bio-Rad). Bands intensities were quantified using ImageJ software (version 1.53k, Rasband). The following antibodies were used: mouse monoclonal anti-Nesprin 2 (1:2000, MA5-18075, Thermo Fisher Scientific), mouse monoclonal anti-GAPDH (0411) (1:5000, sc-47724, Santa Cruz Biotechnology Inc), and goat anti-mouse IgG (H + L)-HRP (1:10000, 115-035-003, Jackson ImmunoResearch).

### 2.12 Statistical analysis

Statistical analysis was performed using GraphPad Prism 10.0. Differences between groups were assessed using either a paired Student’s t-test or a non-parametric signed-rank Wilcoxon test, based on data distribution. The normality of the data was evaluated both visually and with the Kolmogorov-Smirnov test. p<0.05 was considered statistically significant.

## 3 Results

### 3.1 WES germline analysis results

The complete workflow of this study is represented in **Figure 2**. First, WES analysis was performed on 204 patients with a surgical and/or clinical diagnosis of EM enrolled in the study. Overall, 12 patients were identified as carriers of variants in these genes, including six harbouring *SYNE1* (NM_182914.3) variants and six *SYNE2* (NM_182961.4) variants. Notably, two patients (ID 8, 12) were found to carry the same *SYNE2* variant [*i.e.*, c.18001G>A, p.(Asp6001Asn)] despite no familial relationship (**Table 1**). To elucidate the functional consequences of these variants and assess their potential involvement in endometriotic cell migration, we selected these patients carrying rare germline, *in silico*-predicted damaging variants for further investigation.

**Figure 1:**
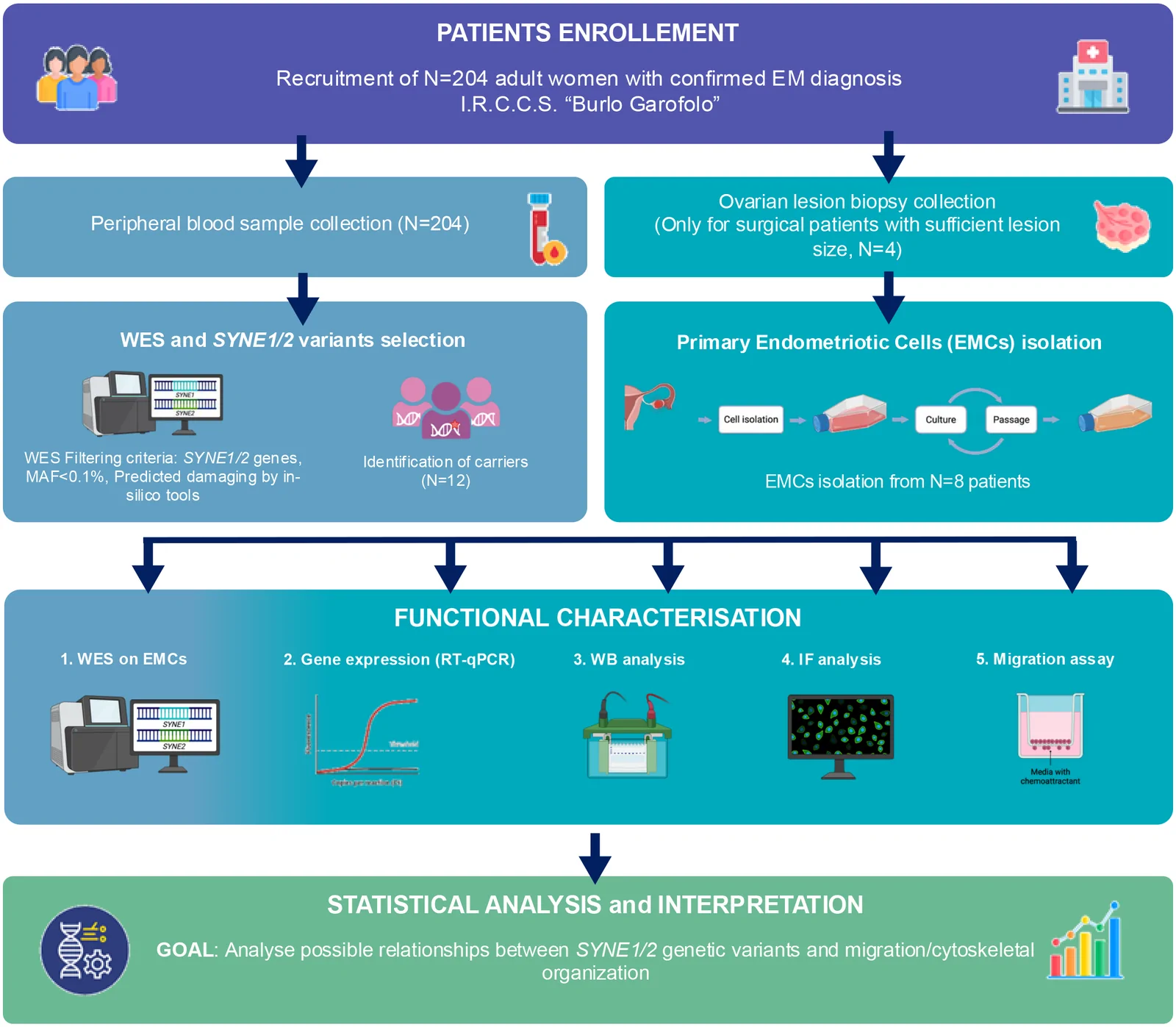
Schematic overview of the study workflow. “Blood samples from 204 EM patients underwent WES for rare *SYNE1/2* variants (MAF <0.1%). Concurrently, primary EMCs were isolated from eight patient biopsies for somatic WES and SYNE2 functional characterization (RT-qPCR, Western blot, immunofluorescence, and migration assays). Statistical analyses evaluated associations between *SYNE2* variants, cell migration, and cytoskeletal organization. (*Icons: Flaticon/BioRender*)”.

**Figure 2:**
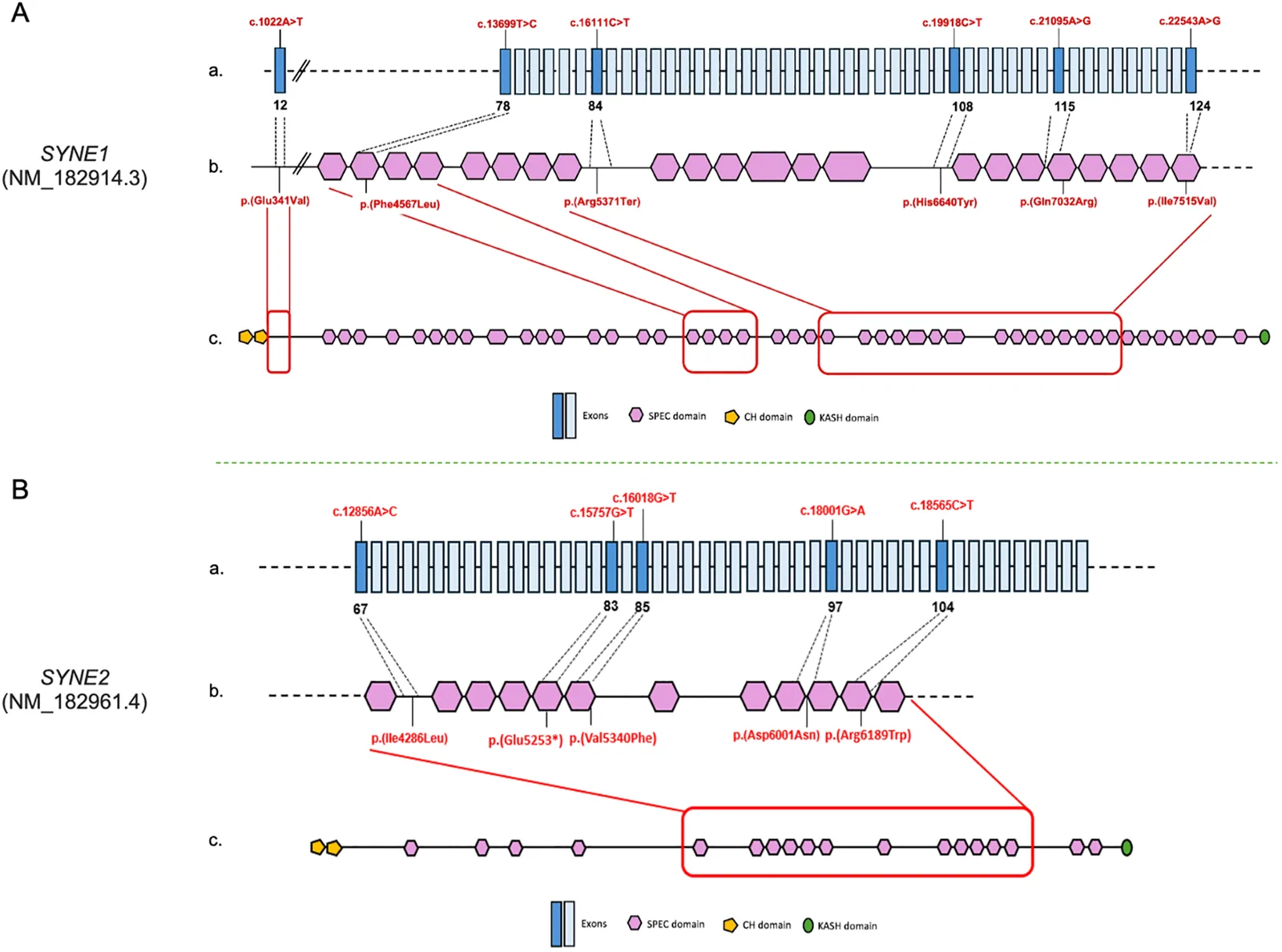
Schematic representation of *SYNE1* and *SYNE2* genes. Schematic representation of *SYNE1* **(A)** and *SYNE2* **(B)** cDNA. **(a)** Light blue boxes indicate exons not affected by damaging variants, while dark blue boxes mark exons that contain the identified variants (in red). Numbers below the boxes correspond to the exon number affected by the variants. **(b)** Zoom of the region of *SYNE1/2* proteins (Nesprin-1 and Nesprin-2, respectively) affected by predicted pathogenic aminoacidic changes (reported in red). **(c)** Diagram of the entire *SYNE1/2* proteins (A, Nesprin-1 and B, Nesprin-2). Purple hexagons represent the SPEC-domains, yellow pentagons represent the CH-domains (Calponin homology domains), and the green circle represents the KASH domain.

**Table 1:** Rare, likely damaging variants within SYNE1, SYNE2 genes identified in the EM patient cohort.

ID	Cell ID	Chr:pos	Gene	HGVS coding	HGVS protein	PaPI	Polyphen-2	SIFT	CADD	gnomAD	ACMG classification	Reference	Surgery	EM stage	Tissue biopsy
77		6:152230647	*SYNE1*	NM_182961.4:c.21095A>G	p.(Gln7032Arg)	0,924	0,811	0,02	NA	0,00438%	VUS (PM2_supporting)	PMID: 37626618	Yes	III	No
68		6:152321363		NM_182961.4:c.16111C>T	p.(Arg5371Ter)	0,988	NA	NA	NA	0,00159%	P (PVS1, PS4, PM2_supporting)	PMID: 37626618	No	III	No
78	EMC55	6:152211540		NM_182961.4:c.22543A>G	p.(Ile7515Val)	0,637	0,599	0,4	21,8	0,00159%	VUS (PM2_supporting)	PMID: 37626618	Yes	IV	Yes
194		6:152239682		NM_182961.4:c.19918C>T	p.(His6640Tyr)	0.999	0.999	0.03	25	0.00062%	VUS (PM2_supporting)	NA	No	III	No
191		6:152488421		NM_182961.4:c.1022A>T	p.(Glu341Val)	0.944	0.872	0.27	23	0.003285%	VUS (PM2_supporting)	NA	Yes	IV	No
200		6:152330986		NM_182961.4:c.13699T>C	p.(Phe4567Leu)	0.998	0.998	0.02	25.1	0.000137%	VUS (PM2_supporting)	NA	No	NA	No
6	EMC18	14:64119442	*SYNE2*	NM_182914.3:c.12856A>C	p.(Ile4286Leu)	0,893	0,882	0,07	NA	0,00289%	VUS (PM2_supporting)	PMID: 37626618	Yes	III	Yes
9		14:64190200		NM_182914.3:c.18001G>A	p.(Asp6001Asn)	0,995	0,995	0,09	24,5	0,01202%	VUS (PM2_supporting)	PMID: 37626618	Yes	III	No
2	EMC22	14: 64152681		NM_182914.3:c.15757G>T	p.(Glu5253Ter)	0,976	NA	NA	NA	0,00000%	LP (PVS1, PM2_supporting)	PMID: 37626618	Yes	IV	Yes
4	EMC26			NM_182914.3:c.16018G>T	p.(Val5340Phe)	0,924	0,916	0,04	NA	0,00285%	VUS (PM2_supporting)	PMID: 37626618	Yes	IV	Yes
19		14:64209966		NM_182914.3:c.18565C>T	p.(Arg6189Trp)	0,988	0,987	0,02	22,5	0,00040%	VUS (PM2_supporting)	PMID: 37626618	Yes	II	No
93		14:64190200		NM_182914.3:c.18001G>A	p.(Asp6001Asn)	0,995	0,995	0,09	24,5	0,01202%	VUS (PM2_supporting)	PMID: 37626618	Yes	III	No

Genome reference build hg38. Chr:pos chromosome and position of the variant. Gene: name of the gene. HGVS coding, protein: cDNA and protein variant reported according to the Human Genome Variation Society (HGVS) guidelines. PaPI, PolyPhen-2, SIFT, CADD: variant effect evaluated via in silico prediction tools. gnomAD: frequency of the variant reported in the gnomAD database. ACMG classification: classification of the American College of Medical Genetics and Genomics to differentiates sequence variants. Reference: PubMed ID of the selected variant. Surgery: Yes/No indicates whether the patient underwent surgery for EM. EM stage: severity of the EM. Tissue biopsy: availability of sufficient tissue biopsy for the functional validation. NA: not available.

**Figure 1** illustrates the schematic representations of the identified *SYNE1* and *SYNE2* variants mapped onto their respective cDNA sequences, along with the predicted damaging amino acid substitutions shown in simplified models of the corresponding protein structures.

### 3.2 *SYNE2* gene expression by primary endometriotic cells

**Figure 3:**
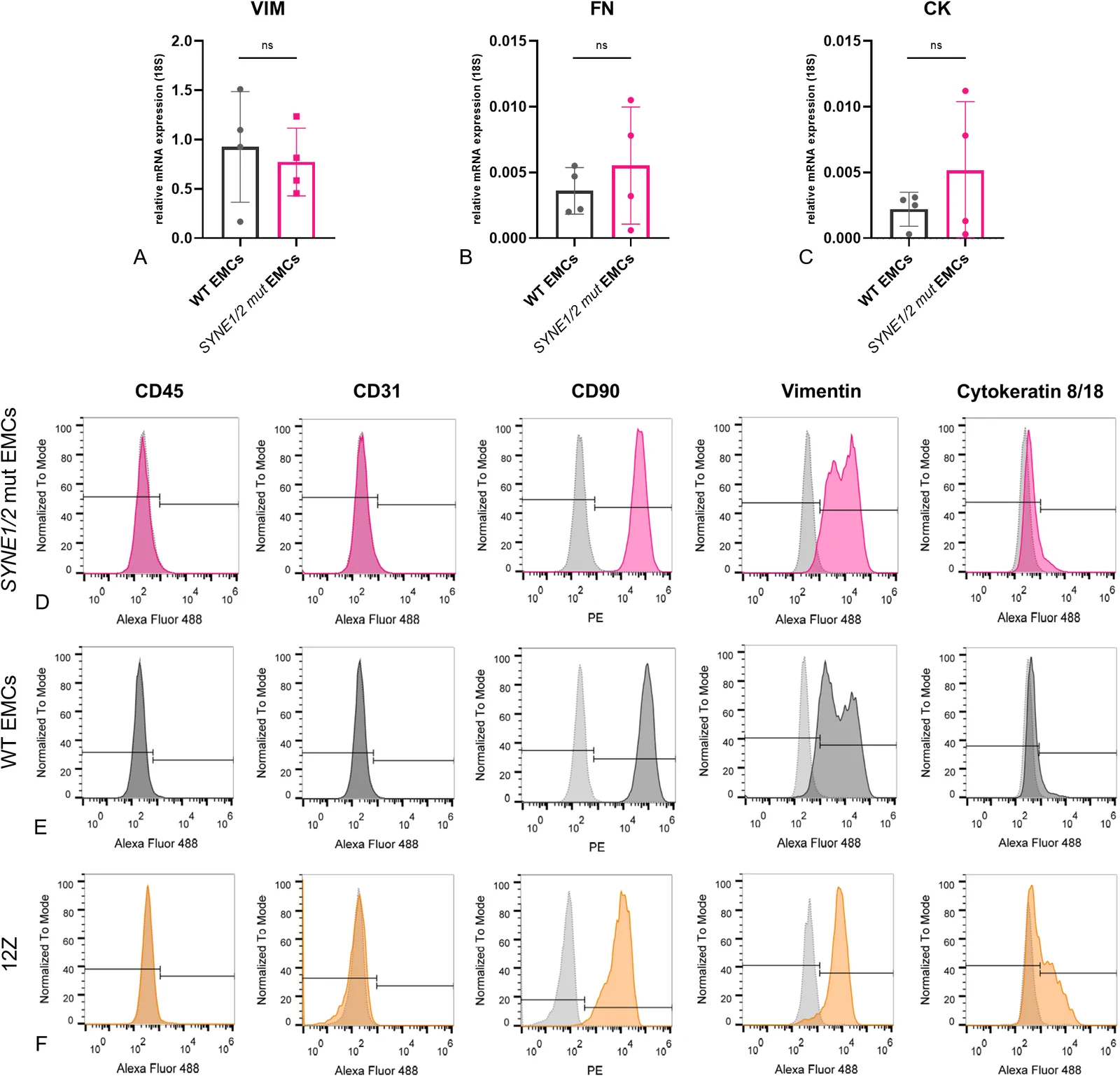
Characterization of EMCs and 12Z via RT-qPCR and flow cytometry. EMC gene expression evaluation of **(A, B)** mesenchymal genes or **(C)** epithelial gene. *SYNE1/2* mut **(D)** or WT EMCs **(E)** or 12Z cell line **(F)** were fixed and stained, after permeabilization (when needed), for the detection of CD45, CD31, CD90, CK8/18, or vimentin. Cytofluorimetric measurements were acquired with AttuneTM NxT Flow Cytometer and analysed with the AttuneTM NxT software. Vimentin, VIM; fibronectin, FN; Cytokeratin 7, CK.

Functional assays were performed to investigate the potential effects of *SYNE1* or *SYNE2* variants on cells isolated from ovarian lesion biopsies (endometriotic cells, EMCs) obtained from EM patients. As outlined in our previous studies, the protocol employed to isolate and culture cells from ovarian lesions yields a heterogeneous population of endometrial and stromal cells that closely resembles the *in vivo* composition of ectopic tissue. We conducted a comprehensive characterization of the EMCs (**Figure 3**; **Supplementary Table 4**) to exclude the presence of contaminating cell types, specifically CD31^+^ endothelial cells and CD45^+^ leukocytes. The mesenchymal and epithelial components of the cell populations were quantified by assessing the expression of mesenchymal markers (fibronectin, FN; vimentin, VIM; CD90) and epithelial marker (cytokeratin, CK) using both RT-qPCR (**Figures 3A–C**) and flow cytometry (**Figures 3D–F**). Our analysis revealed no statistically significant differences between the wild-type (WT) and *SYNE1/2* mut EMCs. In parallel, we characterized an endometriotic cell line named 12Z, which, despite its epithelial histological origin, expresses high levels of CD90 and vimentin, two mesenchymal markers.

Among the 12 previously described *SYNE1/SYNE2* variant carriers, only 9 patients underwent surgery; however, only 4 of them, namely EMC18, EMC22, EMC26, and EMC55 (3 carrying *SYNE2* variants and 1 within *SYNE1* variant, respectively), presented lesions of sufficient size to enable cell isolation procedure (**Table 1**; **Supplementary Table 1**). For comparison, we selected EMC populations from 4 non-carrier patients with available tissue biopsy to serve as WT controls.

Somatic WES analysis was performed on the available EMCs to investigate the presence of additional somatic alterations (“second hits”) affecting *SYNE1/SYNE2* and a list of 22 protein coding genes potentially involved in endometrial cell migration. No additional pathogenic or likely pathogenic somatic variants were identified beyond the previously detected germline heterozygous *SYNE2* variants. Moreover, samples negative for *SYNE2* variants at the germline level also remained negative upon somatic analysis.

**Figure 4:**
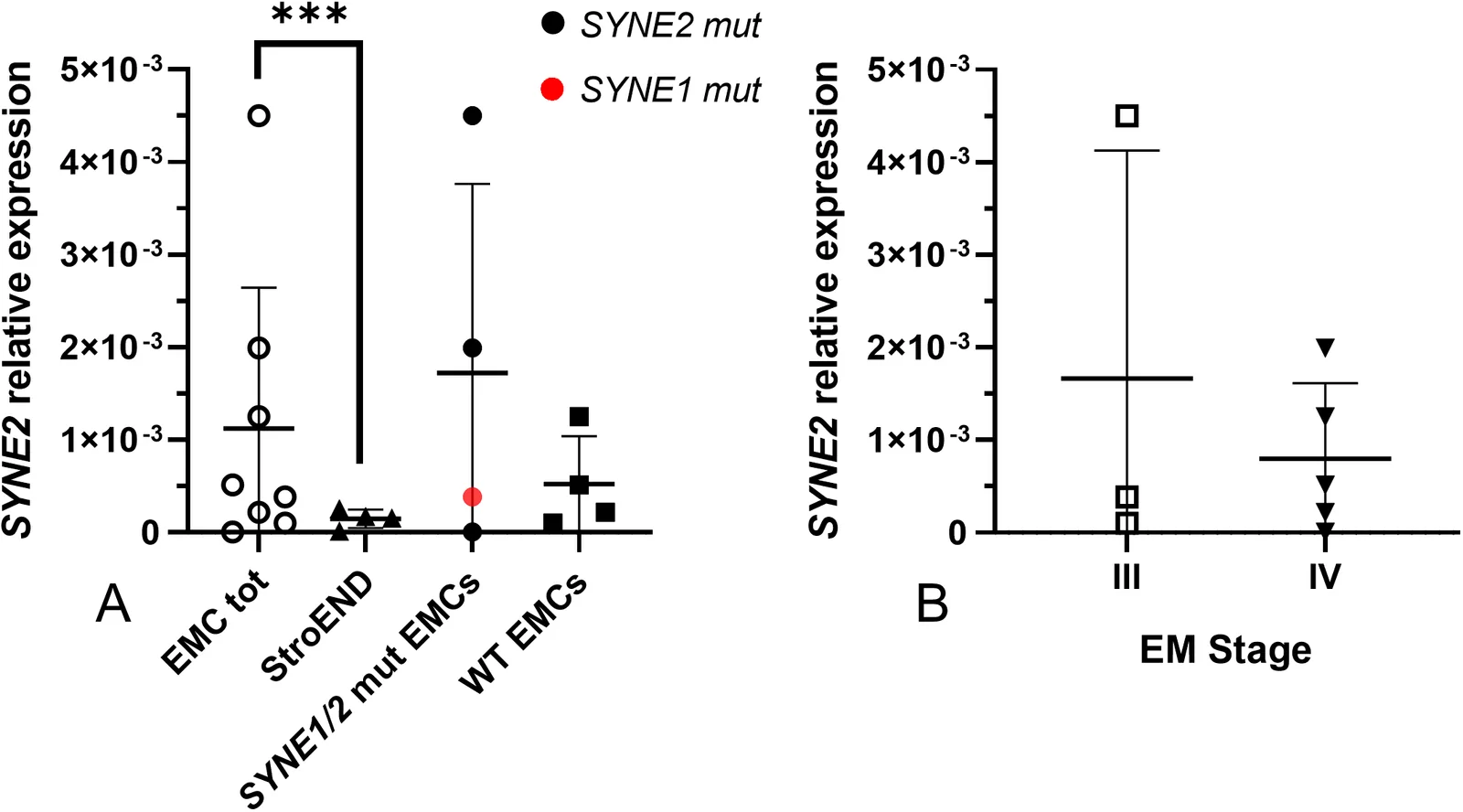
*SYNE2* expression in WT and *SYNE1/2* mut EMCs. **(A)** After total RNA extraction and reverse transcription, *SYNE2* gene expression was analysed by performing RT-qPCR. *RPS18* was used as the housekeeping gene. Scatter plots were generated with the software GraphPad Prism 8.4.3. Empty dots corresponded to all EMCs investigated (n = 8), whereas black dots represented *SYNE2* mut EMCs (n = 3) and red dot *SYNE1* mut EMC population (n =1). F-test indicated a significant difference in terms of data distribution between EMC and StroEND groups (***p < 0.001). **(B)** The clustering of EM patients in stages (III or IV) did not highlight a significant difference in terms of *SYNE2* expression between groups. Data are expressed as mean ± standard deviation (SD).

In order to understand *SYNE1/SYNE2* role in EM development, their mRNA expression was assessed in EMCs using RT-qPCR. Thus, we compared gene expression of *SYNE2* in EMCs, healthy endometrial stromal cells (StroEND), and 12Z (endometriotic cell line of epithelial origin). As shown in **Figure 4**, EMCs exhibited variable *SYNE2* expression levels, which were not correlated with either the presence of *SYNE1/2* variant type (**Figure 4A**) or EM stage (**Figure 4B**). In contrast, *SYNE2* transcript expression in StroEND was more homogeneous (F-test, p<0.001). No *SYNE2* transcript was detected in the 12Z cell line (Data not shown). In addition, we investigated Nesprin-2 protein levels in whole-cell ECM lysates from 4 WT individuals and two individuals carrying *SYNE2* variants. Immunoblotting analysis demonstrated comparable Nesprin-2 protein levels between WT and *SYNE2* mut EMCs (**Supplementary Figure 1**).

### 3.3 Immunofluorescence analysis of Nesprin-2 in endometriotic cells isolated from *SYNE2*-variant carrying patients (*SYNE2* mut EMCs) vs WT endometriotic cells

**Figure 5:**
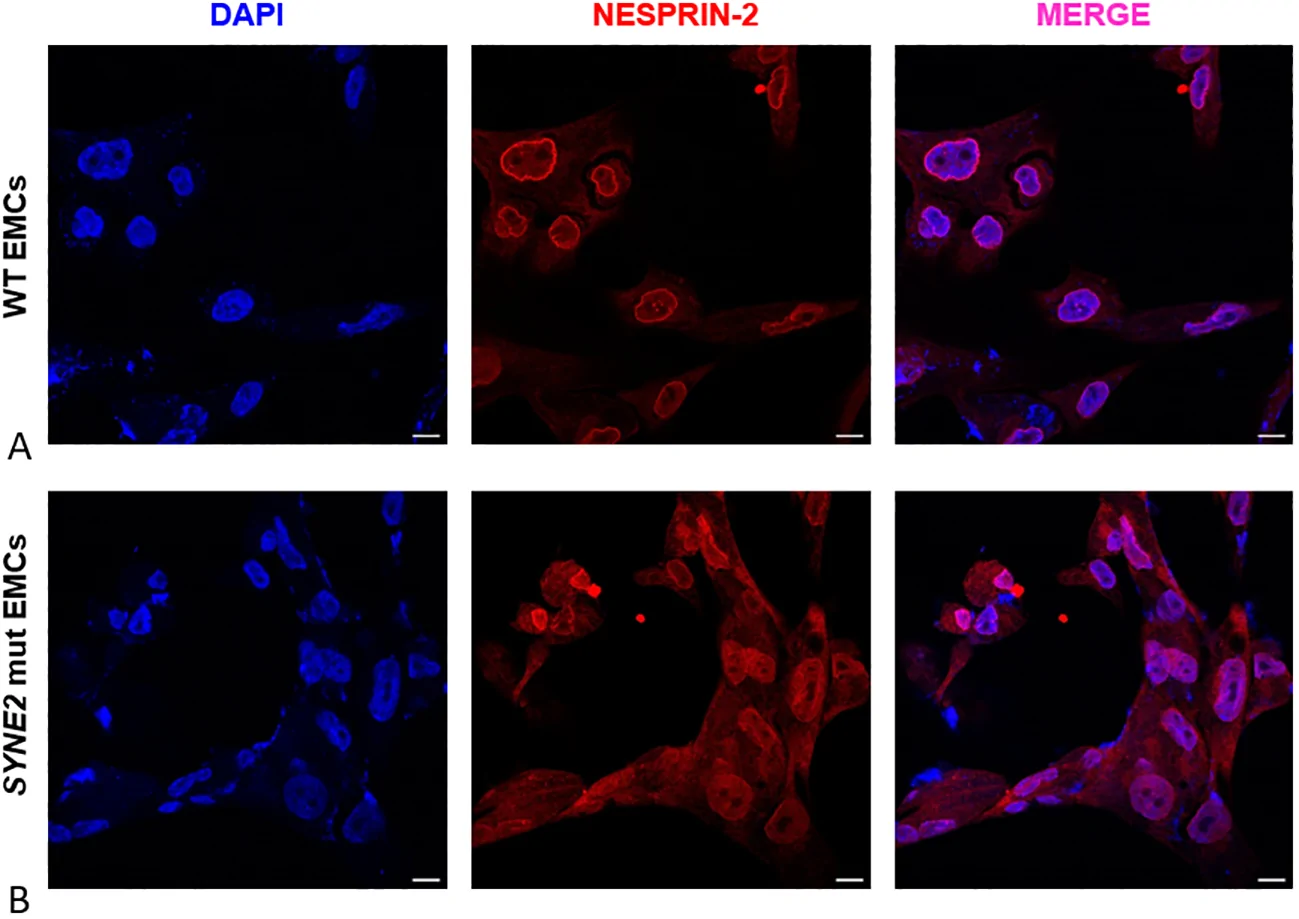
Nesprin-2 localization in WT and *SYNE2* mut EMCs. Representative confocal microscopy pictures of WT **(A)** and *SYNE2* mut **(B)** EMCs stained for Nesprin-2 (red). Nuclei were counterstained with DAPI (blue). Merge images show a predominantly Nesprin-2 signal at the nuclear periphery in WT cells, whereas *SYNE2* mut EMCs display an altered distribution pattern. Scale bar 10 µm.

To investigate whether *SYNE2* variants could alter the synthesis of Nesprin-2 or produce a mislocalization of the protein, we performed immunofluorescence analysis under confocal microscopy on EMCs using a monoclonal antibody against the C-terminal domain of the protein. As shown in **Figure 5**, both WT and *SYNE2* mut EMCs synthesised the protein, but WT EMCs exhibited exclusively nuclear staining, particularly at the nuclear membrane (**Figure 5A**), whereas cells carrying heterozygous variants showed more diffuse staining, including in the cytoplasm (**Figure 5B**). The intensity of this phenomenon varied among SYNE2 mut cell populations but remained consistently present (**Supplementary Figure 2**).

**Figure 6:**
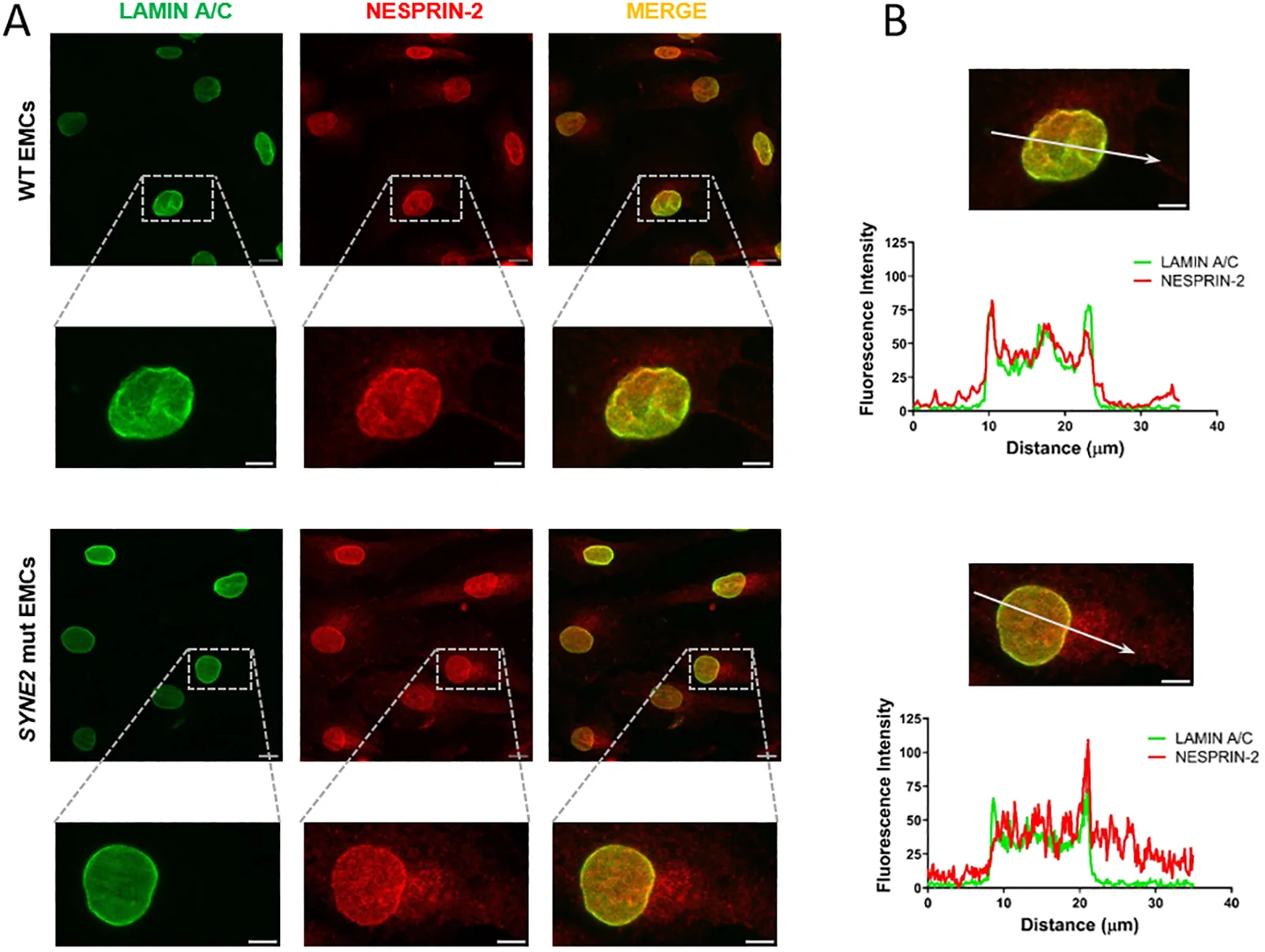
Lamin A/C and Nesprin-2 subcellular organization. **(A)** Representative confocal microscopy pictures of WT (n = 3) and *SYNE2* mut EMCs (n =3) stained for Lamin A/C (green) and Nesprin-2 (red). Merge of fluorescence signals is shown (MERGE). Scale bar 10 µm. Higher magnifications with digital zoom of selected cells are shown, scale bar 5 µm. **(B)** Graphs indicate the fluorescence intensity profile along the grey arrows measured with ImageJ. Analysis shows no evident differences in the spatial organization of Nesprin-2 relative to Lamin A/C, whereas has been confirmed an altered subcellular distribution of Nesprin-2.

**Figure 7:**
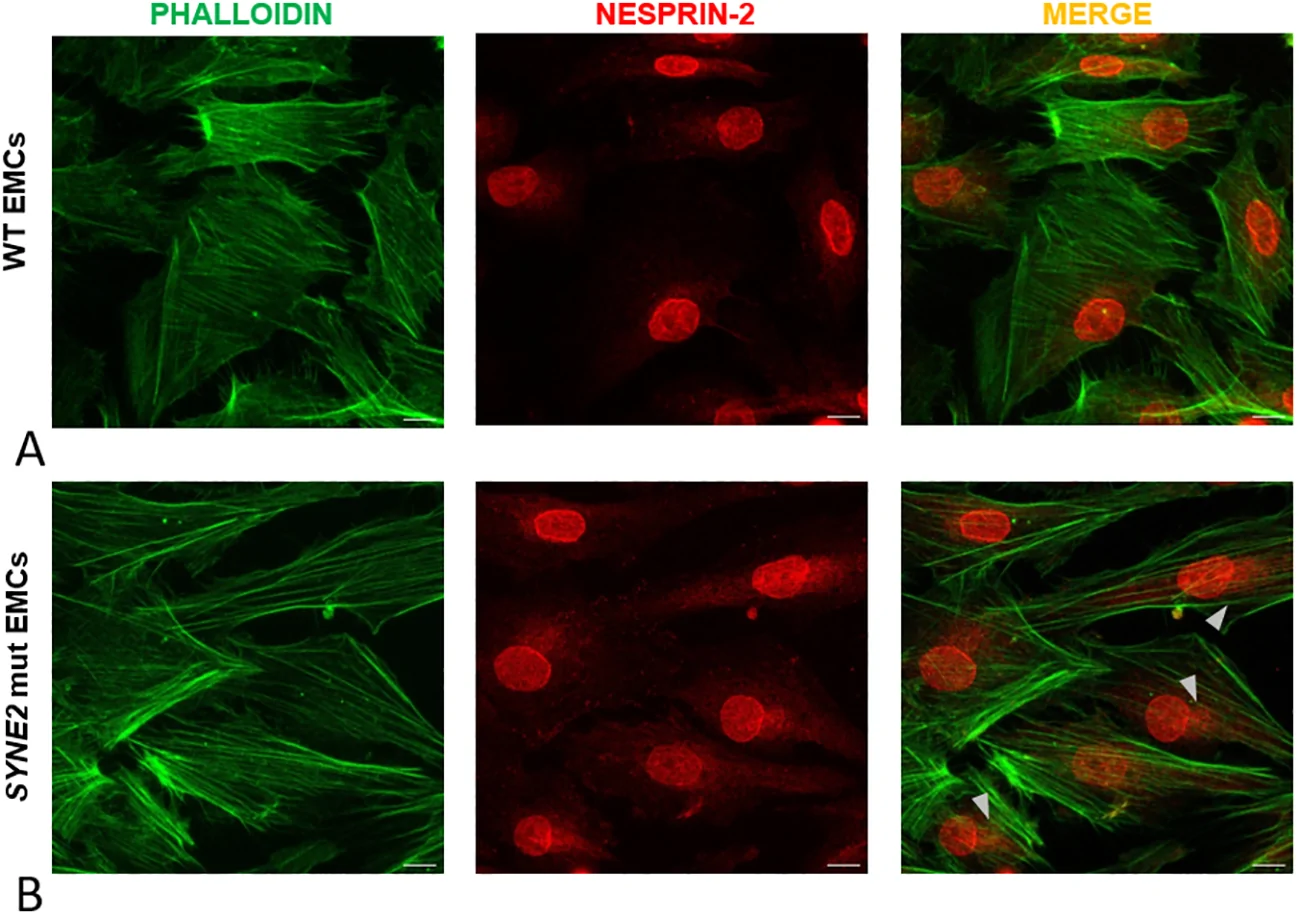
Relationship between Nesprin-2 distribution and actin cytoskeleton organization. Representative confocal images of WT (n = 3, **A**) and *SYNE2* mut (n = 3, **B**) EMCs stained for Nesprin-2 (red) and phalloidin to visualize F-actin (green). Merge of fluorescence signals is shown (MERGE). Grey arrows indicate higher NESPRIN-2 signals along the direction of stress fibres in *SYNE2* mut EMCs, not present in WT EMCs. Scale bar 10 µm.

**Figure 8:**
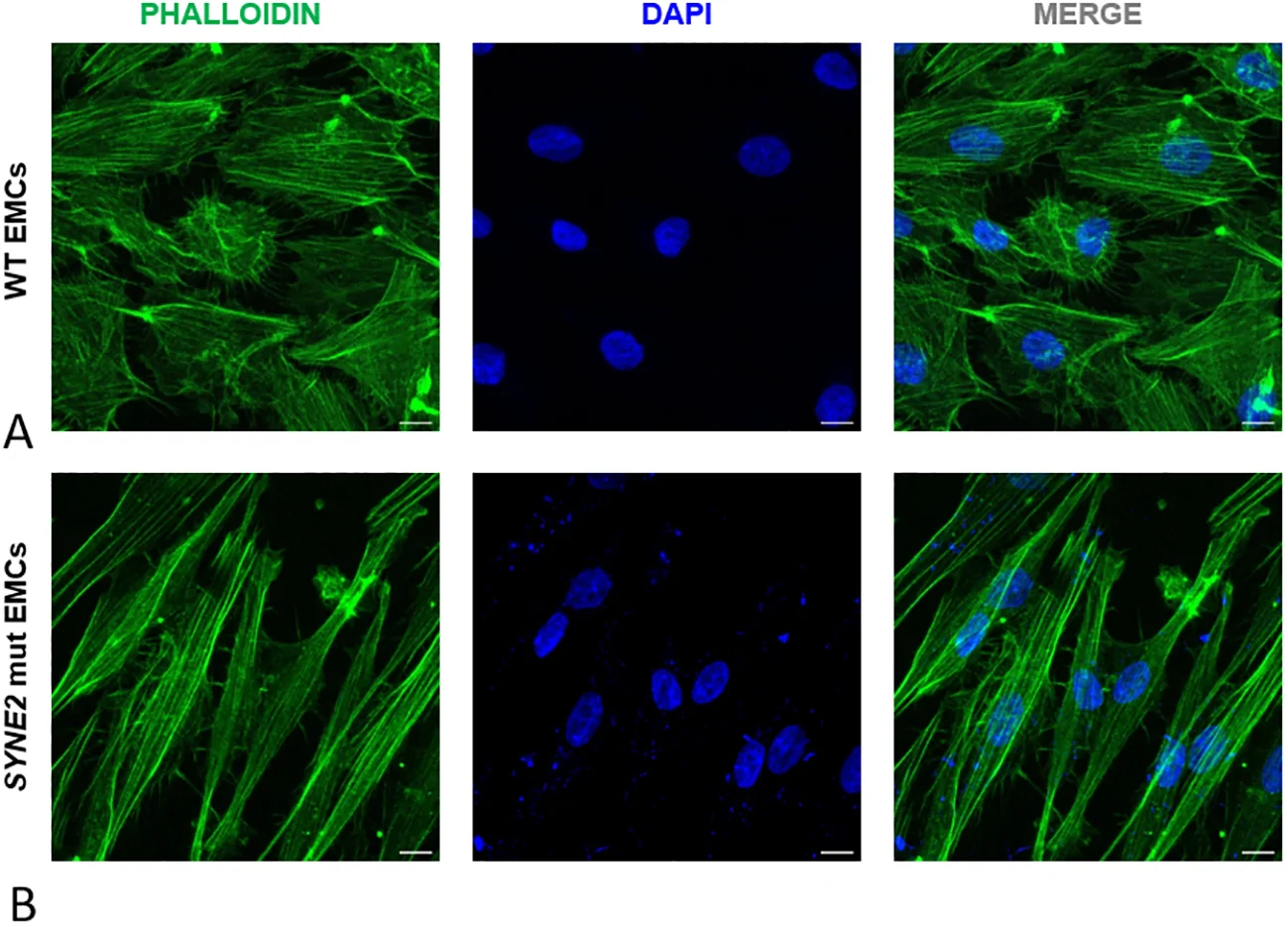
Actin cytoskeleton organization and cell morphology in WT and *SYNE2* mut EMCs. Representative confocal images of WT (n = 3, **A**) and *SYNE2* mut (n = 3, **B**) EMCs stained with phalloidin to visualize F-actin (green) and DAPI to label nuclei (blue). Merge of fluorescence signals is shown (MERGE). Phalloidin staining indicated a more elongated morphology of *SYNE2* mut EMCs. Scale bar 10 µm.

To determine whether these variants affected the interaction with the nuclear lamina, a double staining using rabbit anti-human Nesprin-2 and mouse anti-human Lamin A/C was performed to investigate protein co-localization. Our results showed that the heterozygous variants of *SYNE2* genes did not compromise the interaction of Nesprin-2 with Lamin A/C, as demonstrated in **Figure 6A**. However, double IF analyses highlighted a peculiar staining pattern in *SYNE2* mut EMCs, characterized by a polarized cytoplasmic Nesprin-positive halo. This staining, in fact, seemed to be directed along the cell’s stress fibres. To investigate this observation, we quantified the fluorescence intensity of Lamin A/C and Nesprin-2 in individual cells along a line drawn across the nucleus and extending into the cytoplasm on both sides, oriented along the axis of the actin stress fibres. This analysis confirmed that, in *SYNE2* mut EMCs, Nesprin-2 was also localized in the perinuclear region, with particular intensity along the stress fibre axis (**Figure 6B**; **Supplementary Figure 3**). Finally, to demonstrate the association between actin fibres and Nesprin-2 localization (31), we conducted a further analysis by labelling the WT and *SYNE2* mut EMCs with both fluorescent phalloidin to highlight the microfilaments, and with the anti-Nesprin-2 antibody (32). As shown in **Figures 7A, B**, the directionality of the cytoplasmic Nesprin-2 halo actually run along the actin stress fibres. These investigations also revealed a substantial morphological difference between WT and *SYNE2* mut EMCs: WT cells had a more cuboidal-trapezoidal cell soma (**Figure 8A**), while cells carrying variants had a more elongated shape (**Figure 8B**). This evidence led us to suspect that there could also be differences in the cell migratory capacity.

### 3.4 *SYNE1/2* mut EMCs exhibit increased migratory capacity

**Figure 9:**
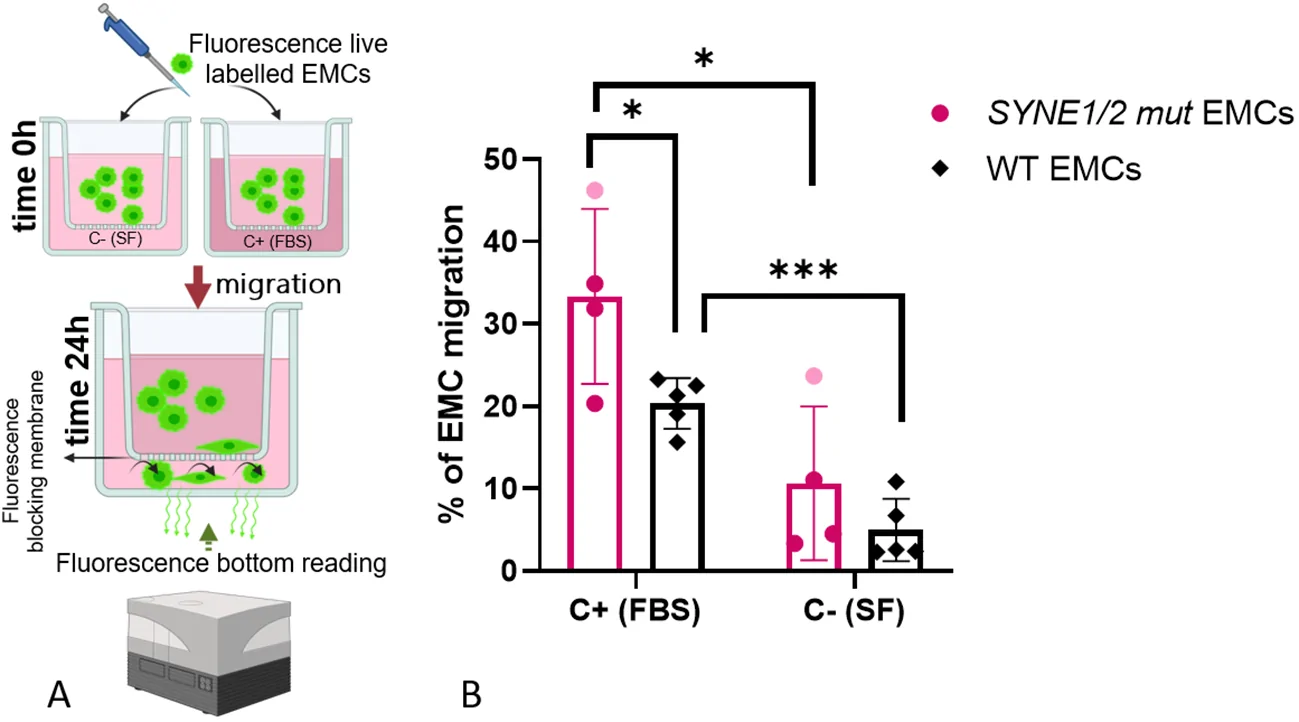
Cell migration analysis of WT versus *SYNE1/2* mut EMCs **(A)** A schematic representation of the migration assay using FluoroBlock Transwells. The experiment was performed as reported in the “Materials and Methods” section using FAST DiI-labelled (1:100 dilution) EMCs. The cells were added to the upper chamber of a transwell system and allowed to migrate through HTS FluoroBlock polycarbonate membranes of 8-μm pore size. The fluorescence exclusively attributable to migrated cells was measured using a Spark Fluorescence Reader in bottom-reading mode and compared with a standard curve generated from different dilutions of labelled cells. **(B)** Percentage of *SYNE1/2* mut EMCs (n = 3 pink, n= 1 light pink the *SYNE1* mut EMC) or WT EMCs (black, n = 5) migration (4 *vs* 5 populations) in the presence (C+) or in the absence (C-) of haptotactic stimuli (FBS). Experiments were conducted in double replicates. Data are expressed as mean ± SD. *p < 0.05, ***p < 0.001.

To understand the functional alterations induced by heterozygous *SYNE1/2* variants, we performed migration assays comparing WT EMCs with *SYNE1/2* mut EMCs. Cells were labelled with a vital fluorescent dye (**Figure 9A**) and were induced to migrate in a double-chamber Transwell (TW) system using foetal bovine serum (FBS) as a haptotatic stimulus (C+). The percentage of cells migrated into the lower chamber of the TW after approximately 24 h was interpolated from a standard curve generated using 100% of the seeded cells (**Figure 9A**). The results obtained demonstrated that the *SYNE1/2* mut EMCs migrated in a greater percentage than WT EMCs, both in the presence of FBS (C+) and under unstimulated conditions (C-). However, in the absence of stimulus, these differences did not reach statistical significance, likely due to the well-known high variability of primary cell behaviour (**Figure 9B**).

## 4 Discussion

EM is a complex and enigmatic gynaecological condition affecting more than 176 million women worldwide (33). To date, the pathogenic mechanisms driving EM have only been partially explored; however, it is increasingly recognised as a disorder with a substantial genetic component and cellular behaviours that resemble tumorigenesis (34). Hence, identifying the molecular players that promote the ectopic implantation and proliferation of endometrial cells is of pivotal importance, as this may facilitate the development of novel diagnostic tools and more effective therapeutic strategies.

Among the several EM gene candidates described over the past decade, GWAS studies have associated *SYNE1* gene with EM susceptibility (21), while reduced expression of *SYNE1* and *SYNE2* has been described in eutopic endometrial tissues from EM women (22, 23). However, the actual role of these genes in EM pathogenesis remains largely speculative. To this purpose, this study aimed to provide novel insights into the functional role of *SYNE1* and *SYNE2* genes in EM onset and progression.

By employing WES analysis, we identified 11 rare, likely damaging germline variants within these genes in 12 EM patients. According to available *in silico* bioinformatic tools, these variants are predicted to alter conserved residues within the Spectrin-repeat domain, which are critical for anchoring the nucleus to the actin cytoskeleton.

To investigate whether additional somatic events could contribute to EM pathogenesis, somatic WES analysis was performed on available patient-derived EMCs. Specifically, we evaluated the potential occurrence of secondary somatic alterations (“second hits”) involving *SYNE1/SYNE2* genes. However, no additional pathogenic or likely pathogenic somatic variants were identified beyond the previously detected heterozygous germline *SYNE2* variants. Furthermore, EMC samples that were negative for *SYNE2* variants at the germline level also remained negative upon somatic analysis. Although these findings could indicate that *SYNE2* alterations in EM are not driven by a classical two-hit tumour-suppressor mechanism, they do not definitively establish a mode of inheritance. Nonetheless, the presence of isolated heterozygous variants is consistent with a potential dominant mechanism, such as haploinsufficiency or a dominant-negative effect, as previously hypothesized for other Nesprin-2-related disorders (32). Additional research involving a larger patients cohort is necessary to clarify the precise inheritance pattern and penetrance of these variants.

We demonstrated through *in vitro* functional assays that these variants could affect nuclear-cytoskeletal dynamics, thus playing a key role in promoting endometrial cell migration. As a first step, we provided evidence of *SYNE2* gene expression in the patient-derived EMCs to functionally validate these variants. Notably, *SYNE2* gene was very low or absent in the healthy endometrial stromal cells (StroEND) and in the immortalized 12Z endometriotic cell line, thus suggesting a possible disease-context-dependent dysregulation of *SYNE2* expression. Our observations showed that *SYNE2* expression levels did not differ among severity stages, although the very low number of patients limits our ability to reach a definitive conclusion. Then, confocal immunofluorescence analysis revealed a clear difference in Nesprin-2 subcellular localisation between WT and *SYNE2* mut EMCs. In particular, in WT cells, Nesprin-2 was located at the nuclear envelope. In contrast, cells derived from carriers of *SYNE2* variants display diffuse cytoplasmic localisation of Nesprin-2, suggesting a failure of proper nuclear envelope anchorage. This alteration in the physiological localisation likely reflects a compromised LINC complex, thereby weakening the structural and mechanical coupling between the nucleus and the cytoskeleton. Hence, this could lead to enhanced migratory capacity of *SYNE2* mut EMCs, as observed in the TW migration assays. This suggests that disruption of Nesprin-2 function may facilitate cytoskeletal reorganization and increase cellular deformability. We exclude that the observed phenomenon could be due to a different epithelial/mesenchymal composition among the isolated EMC populations, as EMC characterization revealed no differences in the expression of epithelial and mesenchymal markers between WT EMCs and *SYNE1/2* mut EMCs.

Members of the WASp family orchestrate a wide range of actin-dependent processes, including cell migration, phagocytosis, endocytosis, and membrane trafficking. Loss of WASp function leads to altered actin polymerization and integrin signalling in immune cells, resulting in impaired homing and directional migration. Furthermore, WASp plays a significant role in the polarization and cytokine secretion of T cells and NK cells (35). Notably, the fact that WASp-associated phenotypes typically arise from homozygous variants, whereas *SYNE*-associated defects manifest in the heterozygous state, suggests that alterations affecting the transmission of mechanical-kinetic stress to the nucleus may be more “sensitive” than those impacting the cytoskeleton proteins. Specifically, alterations that impair the transmission of mechanical forces to the nucleus may exert more pronounced functional consequences than those primarily affecting cytoskeletal organisation itself. Recently, Nesprin-2 was demonstrated as a key mediator of mechanotransduction that inhibits cancer cell growth in the heart (36).

Hence, our functional assays confirm the biological impact of the WES-detected *SYNE2* variants, supporting the hypothesis that disruption of Nesprin-mediated nuclear structure may actively drive the dissemination of endometriotic lesions. This evidence provides a crucial mechanistic bridge between genetic predisposition and somatic cell behaviour, thus paving the way for translating germline genetic data into clinically meaningful insights.

Furthermore, the interplay between Nesprin proteins and other components of the LINC complex (*e.g*., SUN1/2, Emerin) in EM pathogenesis warrants further exploration. Indeed, understanding how these molecular interactions contribute to nuclear mechanics, mechanotransduction, and cytoskeletal organization in endometrial cells may offer critical insights into the mechanisms driving enhanced cell migration, invasion, and lesion establishment. Disruptions in LINC complex integrity could play a pivotal role in facilitating the invasive behaviour of endometrial cells, thereby representing a novel axis for mechanistic and therapeutic exploration in EM.

Despite these promising findings, our study has several limitations. Due to the limited availability of surgically accessible tissue, functional experiments were performed on a relatively small number of patient-derived EMC populations, which may not fully capture the phenotypic spectrum associated with *SYNE1/2* variants. Future studies involving larger cohorts will be instrumental in validating causality and dissecting the precise molecular consequences of these specific variants. Furthermore, the phenotypic and functional impact of these variants directly on the immune system remains to be elucidated. It cannot be ruled out whether the migration abnormalities may also affect leukocytes, potentially contributing to the engraftment of ectopic tissue.

Overall, this study provides evidence that the identification of rare variants in EM may contribute to address the long-standing challenge of the “missing heritability” in EM. Indeed, although GWAS remain a powerful approach for novel gene discovery, rare variants are reported to provide larger effects, thus providing unique insights into the causal mechanism underlying complex disorders (12, 13). Hence, our findings underscore the relevance of extending EM genetic analyses beyond common variants. Moving forward, combining WES data analysis with accurate clinical characterization of patients may represent a novel powerful approach for uncovering genotype–phenotype correlations that can stratify patients by underlying molecular mechanisms in complex disorders.

In conclusion, our work identifies *SYNE1* and *SYNE2* variants as potential genetic drivers of EM and provides the first functional evidence linking Nesprin dysfunction to enhanced migratory capacity in EM. These findings open new avenues for the exploration of nuclear mechanics in EM pathogenesis and suggest that the underlying pathways associated with SYNE1/2 disruption may offer novel insights into risk stratification or highlight potential therapeutic avenues for improving the management of this complex disease.

## Data Availability

The genetic data described in this manuscript have been submitted to the European Variation Archive and are accessible in Variant Call Format at the following link: www.ebi.ac.uk/eva/?eva-study=PRJEB62099.
