# Supplementary material for: The role of SYNE1/2 variants as a potential predisposition factor for the onset of endometriosis
Source: Front Immunol. 2026 Aug 25;17:1888334. doi: 10.3389/fimmu.2026.1888334 (PMC13549908; doi:10.3389/fimmu.2026.1888334)
Supplement: Supplementary file 1 [file DataSheet1.docx]

**Supplementary File**

**The role of *SYNE1/2* variants as a potential predisposition factor for the onset of endometriosis**

**Aurora Santin^1#^, Silvia Pegoraro^2#^, Miriam Toffoli^2^, Giulia Pianigiani^2^, Andrea Balduit^2^, Gabriella Zito^2^, Alessandro Mangogna^3,4^, Paola Trevisiol^3^, Lara Emily Rosso^1^, Federica Mannino^1^, Giovanni Di Lorenzo^2^, Francesca Rossi^2^, Eva Andreuzzi^2^, Chiara Agostinis^2^*, Giuseppe Ricci^1,2^, Roberta Bulla^3^, Giorgia Girotto^1,2^**

^1^ Department of Medicine, Surgery and Health Sciences, University of Trieste, Trieste, Italy

^2^ Institute for Maternal and Child Health - IRCCS "Burlo Garofolo" - Trieste, Italy

^3^ Department of Life Sciences, University of Trieste, Trieste, Italy

^4^ Institute of Pathological Anatomy, Department of Medicine, University of Udine, Italy

^#^ These authors have contributed equally to this work and share first authorship.

**Corresponding author**: Chiara Agostinis ([cagostinis@units.it](mailto:cagostinis@units.it)). Institute for Maternal and Child Health - IRCCS “Burlo Garofolo” - Trieste, Italy. Phone: +39 040 5588652.

**Conflicts of Interest:** The authors declare no conflicts of interest.

**Keywords:** Endometriosis, SYNE2, Whole-Exome Sequencing, Nesprin, migration.

**Supplementary Table 1.**

| **IF** | **Migration** | **RT-qPCR** | **WB** |
| --- | --- | --- | --- |
| 18 | 11 | 18 | 22 |
| 21 | 18 | 21 | 26 |
| 22 | 21 | 22 | 31 |
| 26 | 22 | 26 | 33 |
| 50 | 26 | 45 | 45 |
| 55 | 31 | 50 | 52 |
|  | 33 | 52 |  |
|  | 50 | 55 |  |
|  | 52 |  |  |
|  | 55 |  |  |

**Supplementary Table 1.** EMC populations used for different functional experiments. IF, Immunofluorescence, WB, Western Blotting.

**Supplementary Table 2.**

| **Gene Symbol** | **RefSeq Transcript** |
| --- | --- |
| *CCL18* | NM_002988.4 |
| *CDK6* | NM_001259.8 |
| *CDYL* | NM_004824.4 |
| *CTNNB1* | NM_001904.4 |
| *ESR1* | NM_000125.4 |
| *GPER1* | NM_001505.3 |
| *HOXA11* | NM_005523.6 |
| *KCNN1* | NM_002248.5 |
| *KCNN2* | NM_021614.4 |
| *KCNN3* | NM_002249.6 |
| *MAPK1* | NM_002745.5 |
| *MAPK3* | NM_002746.3 |
| *PAEP* | NM_001018049.3 |
| *PLAU* | NM_002658.6 |
| *POU5F1* | NM_002701.6 |
| *PTEN* | NM_000314.8 |
| *SGPP1* | NM_030791.4 |
| *SNAI1* | NM_005985.4 |
| *SPP1* | NM_001040058.2 |
| *TGFB1* | NM_000660.7 |
| *TLN1* | NM_006289.4 |
| *TWIST1* | NM_000474.4 |

**Supplementary Table 2. Curated list of endometrial cell migration-related genes.** The table lists the 22 genes associated with the search term "endometrial cell migration" extracted from the GeneCards database (https://www.genecards.org/).

**Supplementary Table 3.**

|  | Gene | Tm (°C) | Sense | Sequence (5’🡪3’) |
| --- | --- | --- | --- | --- |
| Ribosomal Protein S18 | RPS18 | 60 | Forward  Reverse | ATCCCTGAAAAGTTCCA  CCCTGTTGGTGAGGTCAA |
| Spectrin Repeat Containing Nuclear Envelope Protein 2 | SYNE2 | 60 | Forward  Reverse | AATCTGCTGCTGTGGTTAGC  ACCAGCTCCTTTTCCAGTTG |
| Vimentin | VIM | 60 | Forward  Reverse | CCAGCTAACCAACGAC  TGCGTTCAAGGTCAAGAC |
| Fibronectin 1 | FN1 | 60 | Forward  Reverse | CGTGGCATTGGGCATT  GGGTGGATATCTCCA |
| Keratin 7 | KRT7 | 60 | Forward  Reverse | TGCACGATCAGACAGTCATTC  GTAGAGCTGAGGAGCGACAATC |

**Supplementary Table 2.** Sequence and characteristics of primers used for RT-qPCR.

| **EMCs** | **%VIM** | **%CK8/18** | **%CD45** | **%CD31** | **%CD90** |
| --- | --- | --- | --- | --- | --- |
|  |  |  |  |  |  |
| SYNE1/2 mut | 95.5 | 15.25 | 0 | 0.25 | 97 |
| WT | 93 | 6.35 | 0 | 0 | 97.5 |
|  |  |  |  |  |  |
| **EMC populations** | **%VIM** | **%CK8/18** | **%CD45** | **%CD31** | **%CD90** |
| 22 | 97 | 19.5 | 0 | 0 | 97 |
| 55 | 94 | 11 | 0 | 0.5 | 97 |
| 45 | 90 | 8.7 | 0 | 0 | 98 |
| 50 | 96 | 4 | 0 | 0 | 97 |

**Supplementary Table 4.**

**Supplementary Table 4.** Percentage positivity of markers expression by EMCs analyzed via Flow Cytometry.

**Supplementary Figure 1**

**Supplementary Figure XX. Nesprin-2 protein levels in primary EMCs derived from WT or *SYNE2*-carrier patients.  (A)** Western blot analysis of Nesprin-2 and GAPDH (loading control) protein expression in whole cell lysates obtained from SYNE2-WT (EMC31, EMC52, EMC33 and EMC45) and *SYNE2*-carrier patients (EMC22 and EMC36) EMC cultures. Band images were acquired using the ChemiDoc MP Imaging System. (**B**) Histograms showing the relative levels of Neprin-2 protein expression. Band intensities were quantified using ImageJ software.

**Supplementary Figure 1. Nesprin-2 protein levels in primary EMCs derived from WT or *SYNE2*-carrier patients**. (A) Western blot analysis of Nesprin-2 and GAPDH (loading control) protein expression in whole cell lysates obtained from WT (EMC31, EMC52, EMC33, and EMC45) or *SYNE2*-carrier patients (EMC22 and EMC26) EMC cultures. Band images were acquired using the ChemiDoc MP Imaging System. (B) Histograms showing the relative levels of Neprin-2 protein expression. Band intensities were quantified using ImageJ software.

**Supplementary Figure 2**

**Supplementary Figure XX. Nesprin-2 protein levels in primary EMCs derived from WT or *SYNE2*-carrier patients.  (A)** Western blot analysis of Nesprin-2 and GAPDH (loading control) protein expression in whole cell lysates obtained from SYNE2-WT (EMC31, EMC52, EMC33 and EMC45) and *SYNE2*-carrier patients (EMC22 and EMC36) EMC cultures. Band images were acquired using the ChemiDoc MP Imaging System. (**B**) Histograms showing the relative levels of Neprin-2 protein expression. Band intensities were quantified using ImageJ software.

**Supplementary Figure 2.**Nesprin-2 localization in *SYNE2* mut EMCs. Representative confocal microscopy pictures of *SYNE2* mut EMCs stained for Nesprin-2 (red). Nuclei were counterstained with DAPI (blue). Merge images show variable alteration of Nesprin-2 distribution pattern. Scale bar 10 µm.

**Supplementary Figure XX. Nesprin-2 protein levels in primary EMCs derived from WT or *SYNE2*-carrier patients.  (A)** Western blot analysis of Nesprin-2 and GAPDH (loading control) protein expression in whole cell lysates obtained from SYNE2-WT (EMC31, EMC52, EMC33 and EMC45) and *SYNE2*-carrier patients (EMC22 and EMC36) EMC cultures. Band images were acquired using the ChemiDoc MP Imaging System. (**B**) Histograms showing the relative levels of Neprin-2 protein expression. Band intensities were quantified using ImageJ software.

**Supplementary Figure 3**

**Supplementary Figure 3. Analysis of Lamin A/C and Nesprin-2 subcellular organization.** Analysis of the fluorescence intensity profile along the gray arrows measured with ImageJ in three different EM WT cells and in three different EMC *SYNE2*-variants cells.
